# Pyrolysis of End-Of-Life Tires: Moving from a Pilot Prototype to a Semi-Industrial Plant Using Auger Technology

**DOI:** 10.1021/acs.energyfuels.4c02748

**Published:** 2024-08-23

**Authors:** Alberto Veses, Juan Daniel Martínez, Alberto Sanchís, José Manuel López, Tomás García, Gonzalo García, Ramón Murillo

**Affiliations:** † Instituto de Carboquímica (ICB-CSIC), C/Miguel Luesma Castán 4, Zaragoza 50018, Spain; ‡ Greenval Technologies S.L, C/Ayala 10, Madrid 28001, Spain

## Abstract

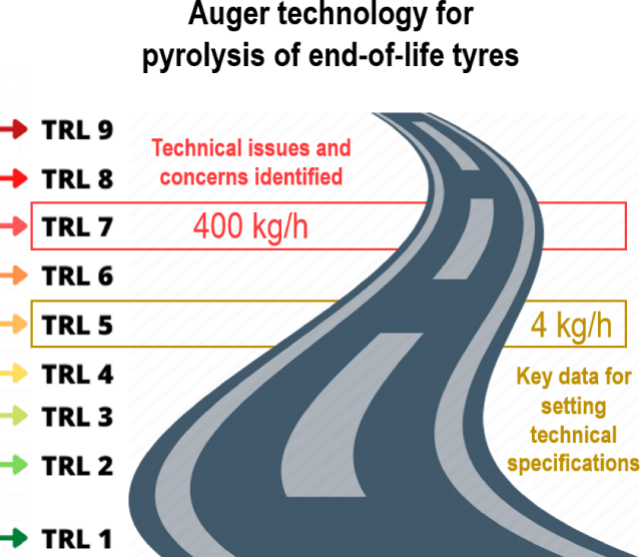

This work, carried out within the framework of the BlackCycle project, demonstrates the robustness of an auger reactor for the pyrolysis of end-of-life tires (ELTs) to be considered within the seventh level of technology readiness (TRL-7). For this purpose, the resulting pyrolysis products are compared with those obtained from a pilot scale facility ranging within the fifth technology readiness level (TRL-5). Using the same type of ELTs, tire trucks (TTs), operating conditions used at the TRL-5 plant are attempted to mimic those expected at a semi-industrial plant: tailored temperature profile (450, 550, and 775 °C) and residence time for vapors (30 s) and solids (15 min). The feed mass rate is 4 and 400 kg/h for the pilot and semi-industrial plants, respectively. The yields of tire pyrolysis oil (TPO), tire pyrolysis gas (TPG), and raw recovered carbon black (RRCB) from both plants, as well as their key properties and characteristics, are in good agreement with each other. The TPO produced by both plants contains comparable concentrations of value-added chemicals such as benzene, toluene, xylene, ethylbenzene, and limonene. There is also a very similar pattern between the simulated distillation curves. The TPG obtained from both plants is also very rich in H_2_ and CH_4_ and has a lower calorific value of 52–54 MJ/Nm^3^ (N_2_ free basis). Although the RRCBs produced by the two plants are more demanding and require more labor, they do have a number of comparable characteristics. All this information demonstrates not only the reliability of the experimental campaigns to scale up the pyrolysis process but also the robustness of the semi-industrial scale plant based on the auger technology to be classified at TRL-7.

## 1 Introduction

Today, end-of-life tires (ELTs) are seen as a great source of value-added chemicals rather than waste, which supposes important savings of raw materials while reducing environmental footprints.^1,2^ These valuable products can be extracted by pyrolysis to recover building blocks embedded in tires. Thus, pyrolysis of ELTs produces tire pyrolysis oil (TPO) and tire pyrolysis gas (TPG), which come from both natural and synthetic rubber contained in tires. In addition, a solid carbonaceous fraction is obtained, which includes the carbon blacks (CBs) used in tire manufacture. According to ASTM standard D8178, this product must be named raw recovered carbon black (RRCB). Once the RRCB has been intensively milled and both steel and fabrics have been removed, it must be denoted as recovered carbon black (rCB). As TPG (15–30 wt %) is primarily used to provide the energy requirements of the process,^3^ marketable products from pyrolysis of ELTs are TPO (35–45 wt %) and RRCB (35–45 wt %). TPO represents a chemical pool not only for the recovery of valuable compounds such as single-ring aromatics and limonene^4−7^ but also as a feedstock in the CB industry.^8^ In general, this complex mixture of aromatic, aliphatic, polar, and heteroatomic hydrocarbons appears to be very attractive as a replacement for various fossil sources in refinery units.^9^ RRCB is expected to play a very interesting role in the substitution of virgin CB as a reinforcing agent in various polymer products, as well as in other applications related to catalysts, and engineered carbons as activated carbon.^10,11−13^

**Figure 1:**
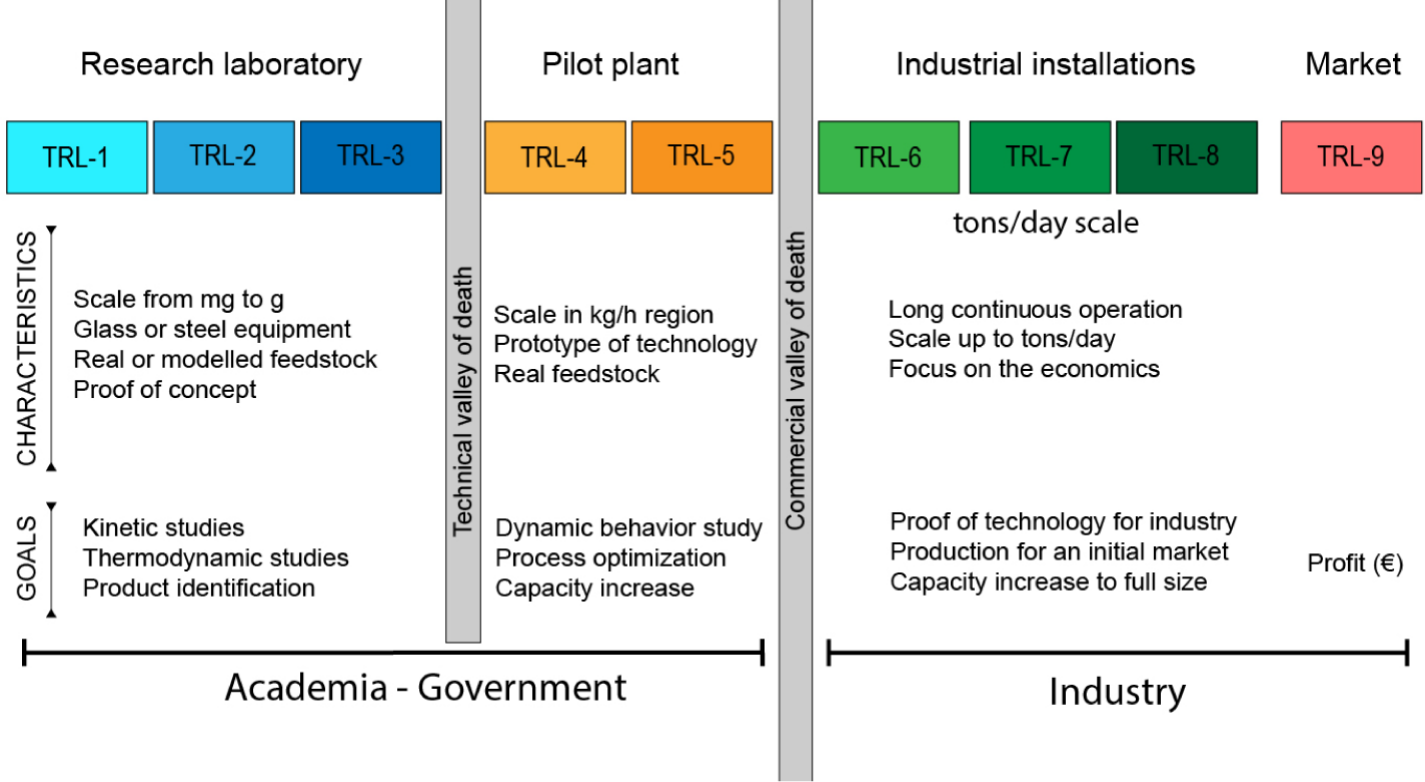
General scheme of the TRL concepts.

Pyrolysis is an ancient process used for centuries to carbonize wood in order to synthesize vegetable charcoal.^14^ The first developments using ELTs as feedstock date back to the 70s with the first oil crisis.^15^ Since then, the pyrolysis of ELTs has been studied worldwide for years, with particular attention being paid to the influence of the governing variables on both the yield and properties of the resulting products using laboratory-scale facilities.^4,16^ At the pilot scale, limited studies are found in the literature.^1,3,17−19,21,22^ In addition, information at the industrial scale is practically nonexistent, and the available data are only found on the websites of technology providers. Technical data on the pyrolysis of ELTs on an industrial scale are certainly scarce in the literature, considering that more than 50 years of intensive research have passed since the first reports. These information gaps are often referred to as the “valley of death”, which is divided into the technical and the commercial (Figure 1). The Environmental Research Group of the Instituto de Carboqumica (ICB-CSIC) has been working for years on the transition between laboratory-scale facilities and pilot plants of thermochemical processes for the valorization and recycling of different types of waste. That experience can also be seen as bridging the technical “valley of death” between technology readiness levels (TRLs) of the formers: from 1 to 3, to 4–5. With this background, and in close collaboration with private companies, this work addresses the challenges associated with the so-called commercial “valley of death” as it attempts to move the pyrolysis of ELTs from a pilot plant (TRL-5) to semi-industrial/industrial scale (TRL-6–9).

Refineries around the world are increasingly interested in alternative feedstock to crude oil, mainly from waste. The ultimate goal is to replace some of their inputs and thus contribute to some extent to defossilization of the chemical sector enabling, the transition from a linear to a circular carbon economy.^23−25^ In this regard, studies showing the potential use of TPO in key refinery units such as distillation,^7,8,26^ fluid catalytic cracking (FCC),^23,27^ and hydrotreatment,^28,29^ among others, are becoming more common. The results obtained in this field are very encouraging with regard to the possibility of blending TPO in low concentrations with conventional crude oil streams to produce-high quality derivative products without altering the required operating conditions.^9^ However, these refinery units operate at very high throughputs, so the amounts of TPO also need to be significant to demonstrate the viability of these routes. Therefore, the development of semi-industrial pyrolysis plants, following the different stages of the TRLs based on the experience gained in research and development (R&D), i.e., on pilot scale prototypes extensively studied, is considered to be an essential step to ensure a truly circular economy for ELTs.

Similarly, RRCB is attracting considerable attention as a replacement for virgin CB in various rubber formulations.^10,30^ As a reinforcing agent, more than 90% of the world’s CB production is used in the manufacture of rubber, particularly tires.^31^ Thus, the recovery of CB from ELTs is seen as a concrete action from a circular economy perspective. It is also worth noting that any black colored product contains CB, and for this and other reasons, the CB market is expected to continue growing in the coming years. The CB industry is characterized not only by the handling of very large quantities of materials but also by the delivery of consistent properties over time and constant production volumes. In this sense, the pyrolysis of ELTs must make progress in the production and characterization of RRCB from semi-industrial plants in order to fill the gap left by pilot-scale prototypes and thus meet the requirements imposed by the sector. In all cases, the RRCB must be converted to rCB in order to meet the ASTM D8178 standard for the best performance as a reinforcing agent in rubber formulations.

The incorporation of both TPO and RRCB into practical applications at the industrial scale promotes pyrolysis as a mechanism for the circular management of complex end-of-life products such as ELTs. This means that TPO and RRCB could offer real technical and economic benefits as well as a reliable contribution to reducing carbon footprints. Both pyrolysis products are expected to contribute to the closing of the loop in the tire and rubber industry. To the best of the authors’ knowledge, there are no works in the literature that focus on the scale-up of the ELTs pyrolysis process, although it should be mentioned that there is very useful information available for biomass.^32^ Studies detailing the operating conditions and real yields and properties of the resulting products on a semi-industrial scale provide an important impetus for pyrolysis to move toward a circular economy for tires. In addition, the challenges associated with the risks posed by exogenous factors such as the dynamics of the market, the reliability of the infrastructure, and the supply chain are expected to be clarified.^33^ The scalability of pyrolysis processes is overcome with reliable data coming from experimental campaigns using semi-industrial plants, as there is no simple scale-up strategy to bring this type of thermochemical process to maturity.^34^

As a step forward, this work describes for the first time in the literature the yields and properties of the products obtained from the pyrolysis of ELTs using two different reactor scales based on the auger technology under similar operating conditions. In this respect, the results obtained at the semi-industrial scale plant (400 kg/h) are sufficiently robust to be classified at the seventh technology readiness level (TRL-7). These results are fully comparable with those obtained from a pilot scale facility (4 kg/h) that has been intensively studied by our research group,^3,35,36,39,43^ and it aligns with the characteristics of the fifth technology readiness level (TRL-5). In particular, the experimental conditions in the TRL-5 facility have been meticulously selected to allow an accurate comparison, including a similar volatile residence time (controlled by the feed rate of 4 kg/h) and the same temperature profile along the reactor (450, 550, and 775 °C). The auger reactor is an emerging and promising technology that offers a small specified reactor size, low carrier gas flow rate, and minimal energy requirements.^44^ Auger reactors also show an interesting versatility in the handling of solids with poor flowability, such as ELTs.^45,46^ They are easy to operate with different arrangements of temperature distribution along the reactor, enabling the integration with different heat transfer media, among others. The results reported in this work are expected to provide a major breakthrough in the circular economy of tires and rubber products, while highlighting the benefits of the auger technology at the semi-industrial scale.

## 2 Materials and Methods

### 2.1 Feedstock: End-Of-Life Tires

Both the pilot plant (TRL-5) and the semi-industrial plant (TRL-7) used granules of ELTs from truck tires (TTs) without steel or textile fibers. However, the particle size of the TTs used in the pilot plant was lower (2–4 mm) than that used in the semi-industrial plant (20–60 mm). This feedstock was provided by Estato Umweltservice GmbH. Table 1 summarizes the characteristics of the feedstock in terms of ultimate and proximate analyses and the higher calorific value (HCV), carried out in accordance with standards UNE-EN 15407 (Thermo Flash 1112), UNE-EN 15402-3 (IKA C-2000), and UNE-EN 15400 (Parr 6400), respectively. The characterization data of the ELT samples reported in Table 1 are found clearly in line with previously published data sets in the literature.^4,5,12^ Proximate analysis is a useful indicator for predicting the final product yields, as the volatile matter is expected to be completely converted to TPG and TPO, leaving fixed carbon and ash in the RRCB fraction.

**Table 1:** ELT Characterization

analysis, as received basis	sample TT
Proximate (wt %)	
moisture (wt %)	1.0
ash (wt %)	5.9
volatile matter (wt %)	65.0
fixed carbon (wt %)	28.1
Ultimate (wt %)	
carbon	84.5
hydrogen	7.11
nitrogen	0.49
sulfur	1.72
Calorific value	
HCV (MJ/kg)	37.5

### 2.2 Plants’ Description

#### 2.2.1 Pilot Plant

The pilot plant used in this work is based on the single-auger technology and has been continuously revamped and used for years by our research group for the pyrolysis not only of ELTs^3^,^35−38^ but also of biomass^40,42,43^ and polystyrene waste.^37^ The plant is located in the laboratories of the Instituto de Carboqumica (ICB) in Zaragoza, Spain. It can process up to 10 kg/h of shredded rubber. The ELTs particles are fed by means of an agitated hopper that can hold approximately 25 kg of rubber. The outer part of the reactor is surrounded by 3 independent electrical resistances, which provide the energy for the pyrolysis process. The reactor also has two outlets to direct the resulting vapors released during pyrolysis to the condenser. Various inert gas (N_2_) inlets are located at strategic points in the reactor to ensure that air intrusions are minimized. The N_2_ flow rate used to maintain the inert atmosphere was set at 550 lN/h, using 6 independent gas mass flow controllers (Bronkhorst model: F-201CV-20K-AGD-22-V) with a flow capacity between 20 and 1000 lN/h of N_2_. This N_2_ stream, which has been experimentally shown to have no significant effect on volatile residence time, yield, or product properties in the range of 300 to 2000 lN/h, helps to prevent volatile accumulation and reduces the risk of undesirable phenomena such as backmixing.

**Figure 2:**
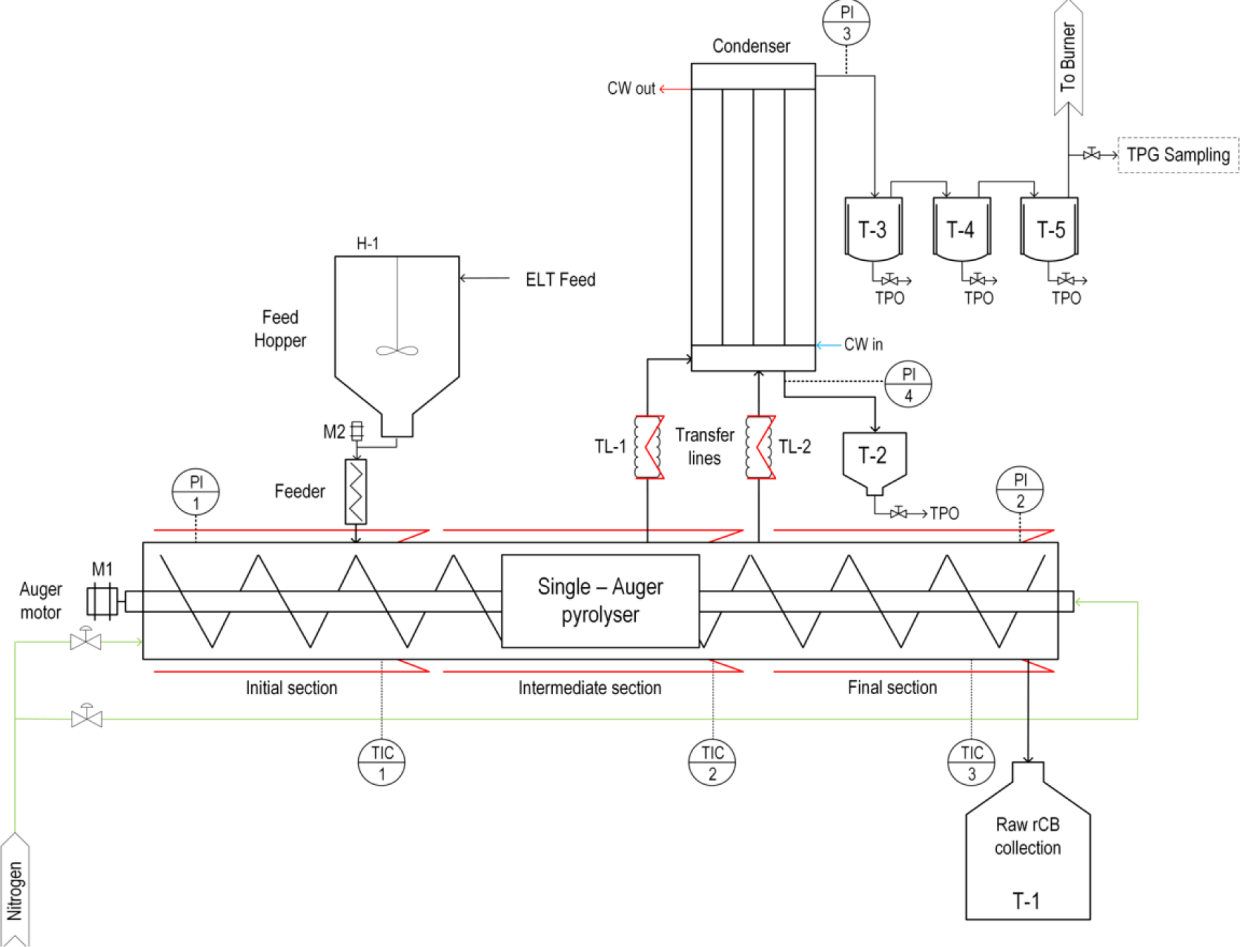
Simplified scheme of the pyrolysis pilot plant.

The condensed stream leaves the condenser by gravity, and it is stored in a small tank that is periodically flushed. The noncondensed gas leaves the reactor through the top and, after expansion to recover liquid droplets, is sent to a flare where it is burned. A sample is taken for GC analysis prior to flaring. The pilot plant is also equipped with a control and acquisition system to control feed rate and solids residence time and to monitor the pressure and temperature at various key points. A simplified scheme is shown in Figure 2. A number of different pyrolysis processes have been successfully carried out using this equipment and more information can be found elsewhere.^41^ It is worth noting that more than 300 h of operation has been accumulated, and more than 1000 kg of TTs has been processed, demonstrating the feasibility and competitiveness of this technology for the pyrolysis of ELTs.

#### 2.2.2 Semi-Industrial Plant

**Figure 3:**
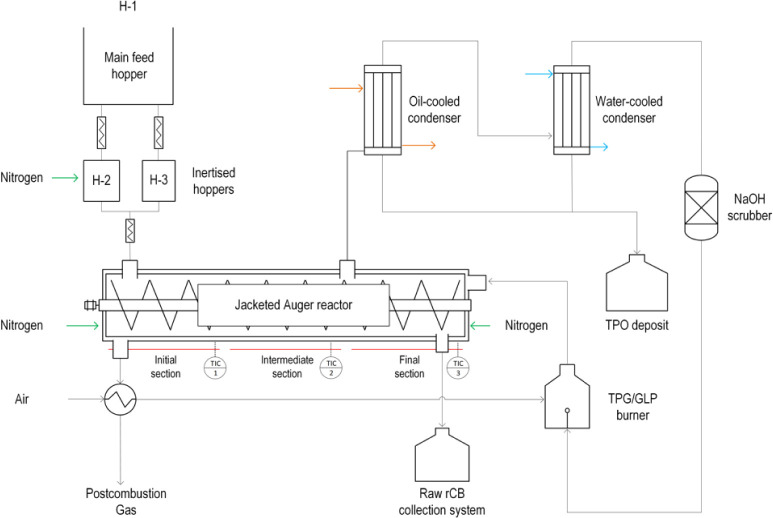
Simplified scheme of the pyrolysis semi-industrial plant.

The semiindustrial-scale plant is located at the “Parque Tecnológico de Reciclado (PTR)” in Zaragoza, Spain. The plant is owned by Greenval Technologies SL, making use of a license from the “Consejo Superior de Investigaciones Científicas (CSIC)”. The Environmental Research Group of the “Instituto de Carboqumica (ICB)” belongs to CSIC. The semi-industrial plant is based on the single-auger technology using the results and operational features of the pilot plant described above. A simplified scheme of the semi-industrial plant is shown in Figure 3. This plant was developed as a prototype to reflect the full-scale system, including all critical components and subprocesses. Extensive testing has been carried out under real operating conditions, including replication of input materials (ELTs) and operating parameters (temperature, mass flow rate, etc.), and this paper presents some of that work. The high throughput, stability, efficiency, and security metrics confirm that the technology operates in a reliable and secure manner, supporting its classification as TRL-7. The reactor geometry of both plants can be considered similar according to the rule of partial similarity based on a dimensional analysis.^34^ This plant was designed for pyrolysis of ELTs at mass flow rates of up to 800 kg/h, i.e., an upscale factor of 80 compared to the pilot plant. The feeding system consists of three hoppers. The first one is used to load the ELTs, and then an endless screw introduces the feedstock into the other two hoppers, which are connected to the pyrolyzer. These hoppers are sealed and inerted under an N_2_ atmosphere. The configuration of these twin hoppers enables a continuous operation, so while one is being filled, the other is continuously feeding the ELTs to the pyrolyzer.

The heating of the auger reactor is provided by the combustion of TPG and supported by an auxiliary liquefied petroleum gas (LPG) burner. The resulting flue gases are routed to an external chamber to provide countercurrent heating, i.e., from the RRCB outlet to the ELT inlet. A temperature profile is then observed along the reactor. N_2_ is used at various points in the plant to prevent possible leaks and blockages caused by the accumulation of gas flows. One of the points of injection is at the solid discharge point to prevent contact of volatile materials with the RRCB. The N_2_ flow rate is controlled by manual valves and an electronic flow meter. The TPO is recovered by two condensers and an expansion system that removes the oil droplets present in the gas. The TPG is compressed and stored in a gas tank that fed the burner. It should be noted that the experimental campaign carried out in the semi-industrial plant lasted more than 100 h of operation and a total of 28 tonnes of ELTs were processed.

### 2.3 Characterization of Products

#### 2.3.1 Tire Pyrolysis Oil

The elemental composition of TPO derived from both plants was determined from ultimate analysis using the UNE-EN 15407:2011 standard (Thermo Flash 1112). The HCV was also measured according to the UNE-EN 15400:2011 standard (Parr 6400). In addition, pH and the total acid number (TAN) were determined using a Mettler Toledo T50 analyzer. TPO was also characterized in terms of density (by gravimetry), viscosity (using a Brookfield LVDV-E apparatus following the standard ASTM D445), and water content (Crison Titromatic, ASTM E203). It should be noted that the results shown in this study are summarized as the average of at least 5 measurements to ensure adequate reproducibility.

In addition, the boiling point distribution was determined according to ASTM D2887 standard using a PerkinElmer Clarus 590 gas chromatograph (GC) equipped with an on-column injector (POC), a wide-range FID detector, and a 10 m Elite-2887 column (0.53 mm ID and 2.65 μm df). An initial oven temperature of 45 °C was maintained for 2 min. A heating rate of 15 °C/min was then applied to reach a final oven temperature of 325 °C. This temperature was maintained for 15 min. The carrier gas was He at a constant column flow of 7 mL/min. The POC injector followed a temperature program of 5 °C above the oven temperature, and the wide-range FID temperature was set at 350 °C. Sample volume injected was 0.5 μL in splitless mode using an auto sampler. The ASTM D2887 quantitative calibration mixture containing C_6_ to C_44_*n*-paraffin was injected to obtain a correlation curve between retention time and boiling point.

A quantification of some relevant compounds present in TPO was also carried out by GC the same instrument as for that the boiling point distribution but with a different configuration. Benzene, toluene, ethylbenzene, *o*-xylene, *m*-xylene, *p*-xylene, and limonene were detected and quantified. A wide-range FID detector and a 60 m DB-5 ms capillary column (0.25 mm ID and 0.25 μm df) were used. An initial oven temperature of 40 °C was maintained for 1 min. A heating rate of 5 °C/min was then applied to reach a final oven temperature of 290 °C. The carrier gas was He at a constant column flow rate of 1 mL/min. The split/splitlee injector and wide-range FID temperatures were 300 and 325 °C, respectively. The sample volume injected was 0.5 μL using an autosampler and a split ratio of 1:30. A quantitative calibration mixture containing 0.2 mass % each of benzene, toluene, ethylbenzene, *o*-xylene, *m*-xylene, and *p*-xylene (BTEX) and 0.26 mass % of limonene was injected to obtain the response factors of the FID detector for each compound.

The TPO from the pilot plant (TRL-5) and the semi-industrial plant (TRL-7) was also separated into light and heavy fractions at atmospheric pressure using a laboratory distillation unit. A flask containing 250 mL of TPO was gradually heated from room temperature to 235 °C. The temperature of the vapor phase was measured with a specially placed thermocouple. The condensed vapors were collected in a flask located after the cooling system to obtain the light fraction (LF). The heavy fraction (HF) was collected as the residual oil in the original flask. The resulting fractions obtained after this fractionation were later characterized for BTEX and limonene compounds according to the procedures described above.

#### 2.3.2 Raw Recovered Carbon Black

The RRCB was characterized by ultimate and proximate analyses and calorific value determination using the same procedures as indicated for the TTs. The volatile matter content is an important indicator of the presence of carbonaceous deposits on the surface of the carbon particles as these residues appear to promote the formation of hard agglomerates that severely degrade the quality of the RRCB. Particular attention has therefore been paid to this parameter. The RRCB was also characterized for BET surface area using a Micromeritics ASAP 2020 instrument according to ISO 9277 standard, with an instrument accuracy of 0.01 m^2^/g. The BET surface area is calculated from the physisorption of N_2_ up to a relative pressure of 0.3. The transmittance of the toluene extract was also determined as it is one of the few analytical techniques for virgin CB that has been approved for characterizing RRCB. It was determined in a PerkinElmer Lambda 25 UV/vis spectrophotometer following the ASTM D1618-18 standard.

#### 2.3.3 Tire Pyrolysis Gas

The permanent gases were analyzed on a Bruker 450 GC equipped with a TCD detector. Separation was performed on two SS packed columns in series (Molsieve 13X, HayeSep Q). An initial oven temperature of 60 °C was maintained for 10 min. The carrier gas was Ar at a column flow rate of 30 mL_N_/min. The detector temperature was set at 200 °C. Light hydrocarbons (C_1_–C_4_) were quantified in a PerkinElmer Clarus 590 GC equipped with a flame ionization detector (FID). Separation was performed using a 30-m long and 0.32 mm wide alumina-chloride capillary column. Permanent gases analyzed included hydrogen (H_2_), carbon dioxide (CO_2_), oxygen (O_2_), nitrogen (N_2_), and carbon monoxide (CO), while light hydrocarbons included methane (CH_4_), ethane (C_2_H_6_), ethylene (C_2_H_4_), propane (C_3_H_8_), propylene (C_3_H_6_), isobutane (C_4_H_10_), *n*-butane (C_4_H_10_), *trans*-2-butene (C_4_H_8_), 1-butene (C_4_H_8_), isobutene (C_4_H_8_), *cis*-2-butene (C_4_H_8_), and 1,3-butadiene (C_4_H_6_). Sulfur compounds were analyzed on a PerkinElmer Clarus 590 GC equipped with an FPD detector. Separation was performed using a 30 m Rt-Silica BOND capillary column. An initial oven temperature of 40 °C was maintained for 2.5 min. A heating rate of 15 °C/min was then applied to reach a final oven temperature of 180 °C. This temperature was maintained for 2 min. The carrier gas was He at a constant column flow rate of 2 mL_N_/min. The injector and FPD temperatures were 250 and 300 °C, respectively. Sulfur compounds analyzed included carbonyl sulfide (COS), hydrogen sulfide (H_2_S), carbon disulfide (CS_2_), and methyl mercaptan (CH_4_S). Certificated gas mixtures (Air Products) were used for identification and quantification purposes.

## 3 Results and Discussion

### 3.1 Pyrolysis Conditions

Both plants were operated under very similar conditions in terms of the controlling variables involved in auger pyrolyzers: temperature and residence time of solids and vapors in the reactor.^44−49^ As hot gas was used to heat the semi-industrial plant, a temperature profile was observed along the reactor as the TTs are converted. On the other hand, the pilot plant was equipped with three independent electrical resistances to provide the energy required for pyrolysis. A temperature profile similar to that observed in the semi-industrial plant was therefore set. The temperatures observed for both plants in the first, middle, and last sections of the reactor for both plants were 450, 550, and 775 °C, respectively, and the residence time of the rubber particles in the reactor was 15 min. Based on previous tests carried out by our research group, this temperature profile had the added benefit of maximizing TPO production while ensuring a low volatile matter content in the RRCB.^38^ The temperature was measured using a series of thermocouples placed on the inner walls of the lower part of the reactor. This allowed the temperature to be approximated by the temperature of the ELT particles.

**Figure 4:**
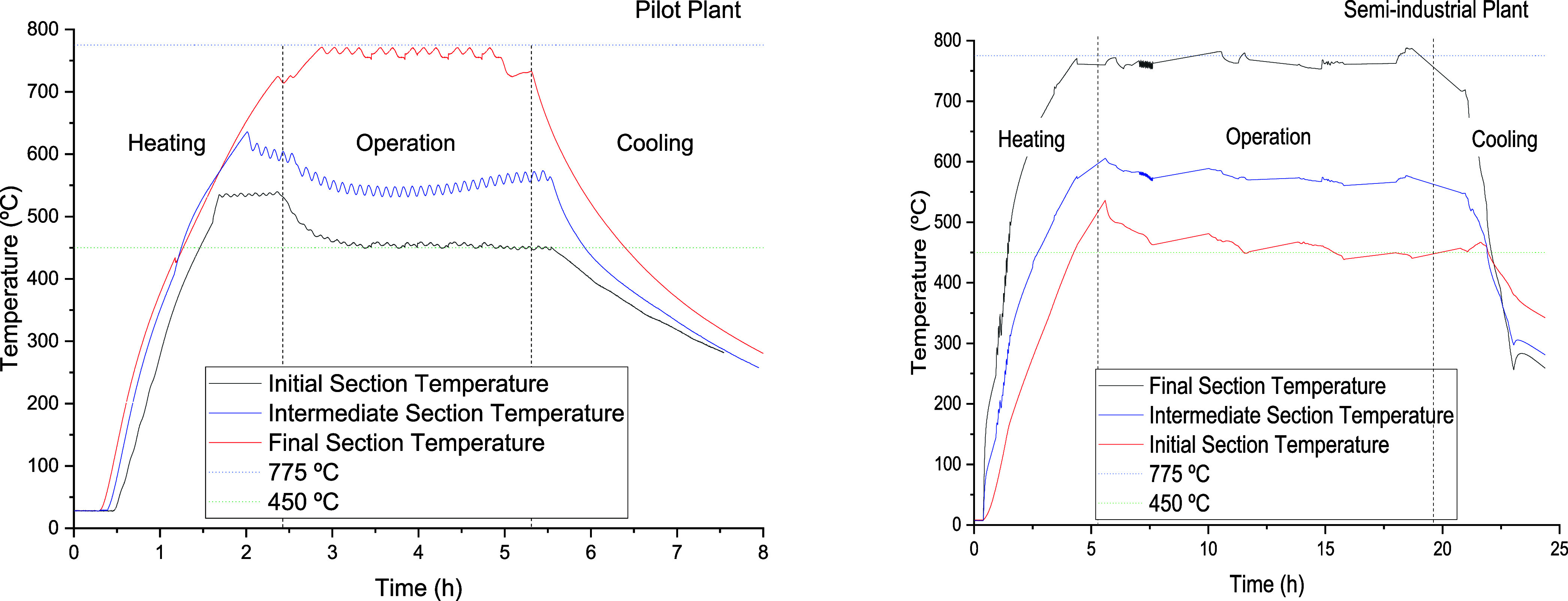
Temperature profile: a) pilot plant and b) semi-industrial plant.

Figure 4 shows the measured temperature in both plants, which can be considered similar in both the initial and final sections of the reactor. In addition, three steps can be distinguished. The first step corresponds to the heating of the reactor. The temperature in the first section is set a few degrees higher than expected (450 °C). Once the feedstock is introduced into the reactor and the pyrolysis process takes place, this temperature decreases until the desired temperature is reached. This process takes about 30–45 min in the TRL-5 plant and 12–14 h in the TRL-7 plant. The second step begins after this period, when a steady state with no significant temperature variations is reached in both plants. The final and third steps are the temperature drop profile. This step takes place after 10 and 24 h of testing for the pilot plant and the semi-industrial plant, respectively; and means that no more energy is being supplied, and the test can be considered complete.

On the other hand, the mass flow rate of the feedstock is the main operating parameter governing the residence time of the vapors in the auger reactor.^50^ The geometry of the reactor can therefore be sued to estimate the degree of filling of the reactor and the residence time of the vapors released during pyrolysis, once their density has been calculated. For this purpose, an internal Aspen Hysys model was used to determine the specific volume occupied by the volatile fraction considering both condensable and noncondensable hydrocarbons inside the reactor. Comparable vapor residence times of about 30 s were found in both plants using mass flow rates of 4 and 400 kg/h of ELTs in the pilot and semi-industrial plants, respectively.

### 3.2 Yields

**Figure 5:**
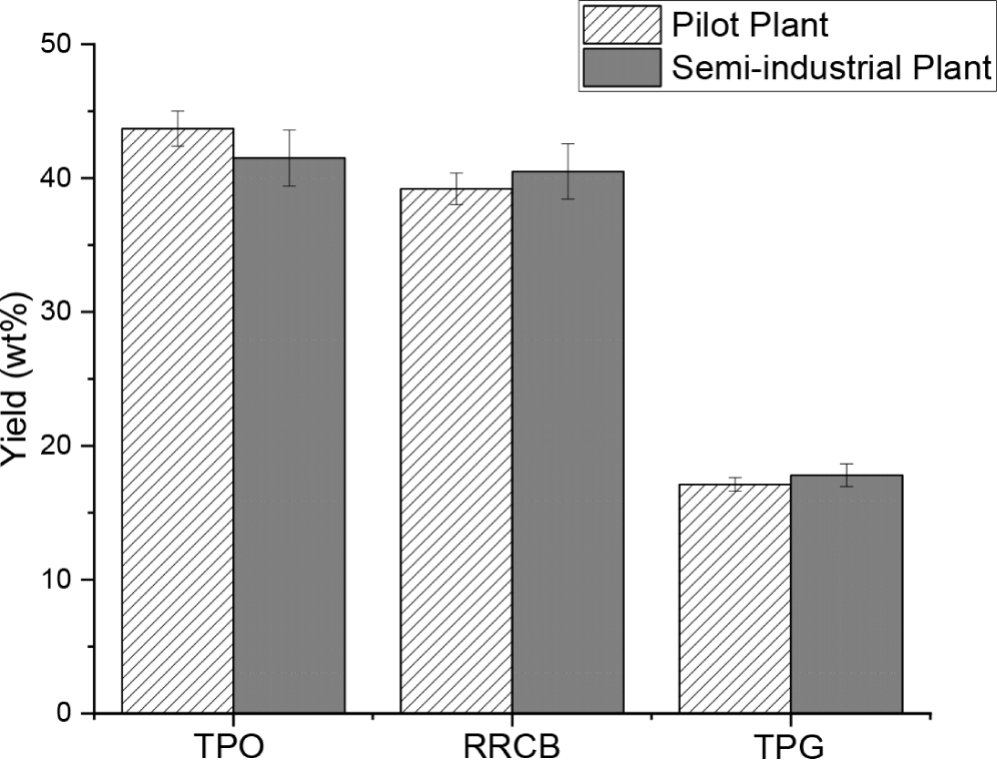
Pyrolysis yields.

Under the above operating conditions, the resulting yields from the pilot and semi-industrial plants for TPO, RRCB, and TPG were determined to be 43.7 ± 2.2 and 41.5 ± 4.3 wt %, 40.5 ± 2.1 and 41.5 ± 4.2 wt %, and 16.2 ± 0.8 and 17.6 ± 1.6 wt %, respectively (Figure 5). These values were calculated as the average of 3 identical experiments. It is worth noting that the resulting yields from both plants are quite similar and serve to demonstrate that the semi-industrial plant works properly in an operational environment. These yields were those expected under intermediate pyrolysis conditions, i.e, when the heating rate of the particles and the residence time of the vapors were around 100 °C/min and 30 s, respectively. A notable advantage of intermediate pyrolysis is its ability to handle a wide variety of feedstock (coarse, shredded, chopped, or finely ground materials), providing versatility and flexibility compared to other pyrolysis conditions.^51^ Similar yields have been reported in other pyrolysis systems using throughputs between 4 and 100 kg/h of ELTs such as rotary kilns^1,20^ and auger reactors.^3,35,38,44,52−55^

It is well-known that temperature is probably the key parameter in pyrolysis, as it plays a very important role not only in the depolymerization of the ELTs but also in the occurrence of secondary reactions, especially at high values. On the one hand, the low temperature in the pyrolysis of ELTs results in some unconverted rubber being fixed in the RRCB, seriously affecting its quality and marketability. At low temperatures, the TPO composition is expected to be very rich in primary pyrolysis compounds, such as limonene. In contrast, high temperatures generally favor the separation of rubber from the carbonaceous solid matrix (depolymerization), and the RRCB is expected to consist mainly composed of carbon derived from CBs. In addition, the TPG yield is increased at the expense of the TPO yield, which is very rich in aromatic compounds due to the promotion of secondary reactions. The temperature profiling technique used in this work takes advantage of both low and high temperatures. This strategy minimizes vapor phase cracking reactions in the first heating section of the reactor prior to volatile evacuation, while subjecting the RRCB to high devolatilization severity in the final heating section.

### 3.3 Properties of the Tire Pyrolysis Oil

Table 2 shows the main properties of the TPO obtained from the pilot and semi-industrial plants. As observed, the results are very similar, and there does not seem to be a major effect on the size of the plant. For both samples, the carbon and hydrogen contents show good agreement, while the sulfur and nitrogen contents are slightly higher in the TPO from the semi-industrial plant. Nevertheless, these concentrations are expected in TPO^4,5^ and are due in part to the presence of sulfur- and nitrogen-containing compounds such as thiophene, benzothiazole, benzothiophene, and benzonaphthothiophene, as well as benzothiazole and benzonitrile, respectively.^26^ Sulfur and nitrogen compounds in the TPO are attributed to some additives used in the vulcanization and formulation of tires.^56,57^ Based on the above results, both TPOs can be considered as a mixture of pure hydrocarbons and hydrocarbons combined with nitrogen and sulfur.

**Table 2:** TPO Characterization

element/property	pilot plant	semi-industrial plant
carbon (wt %)	88.7 ± 0.3	87.5 ± 0.3
hydrogen (wt %)	10.2 ± 0.3	11.3 ± 0.3
nitrogen (wt %)	1.4 ± 0.0	0.6 ± 0.0
sulfur (wt %)	0.76 ± 0.04	0.96 ± 0.04
H/C	1.4 ± 0.03	1.5 ± 0.04
HHV (MJ/kg)	42.3 ± 1.2	41.5 ± 1.2
density at 25 °C (kg/m^3^)	975.7 ± 60	960.0 ± 60
viscosity at 40 °C (mPa.s)	7.0 ± 0.5	5.6 ± 0.5
pH	6.9 ± 0.1	7.3 ± 0.1
TAN (mg KOH/g)	6.2 ± 0.5	5.4 ± 0.5
water (ppm)	253 ± 20	170 ± 20

As expected, the high carbon and hydrogen content gives TPO a remarkable HCV, comparable to crude oil from petroleum (40–42 MJ/kg). It is also worth highlighting the renewable content embedded in the TPO given the presence of natural rubber in the ELTs. TPO is therefore considered to be both a waste-based and renewable hydrocarbon liquid feedstock. Density and viscosity are not only very similar between the TPO produced in the two plants but also show interesting values when compared with those of petroleum-based fuels. These similarities have led to the widespread use of TPO as an alternative to various fuels in a variety of energy systems such as furnaces,^58^ boilers,^59^ and even internal combustion engines.^60,61^ In this sense, motivating results have been reported despite the challenges associated with sulfur, nitrogen, flash point, final distillation point, and polycyclic aromatic hydrocarbons, among others.^26,62^ Interest in the production of transport fuels from TPO has also been on the rise, and the results are very encouraging and interesting.^7,63,64^

However, the use of TPO for the production of chemical commodities appears to be gaining tremendous traction in achieving a more circular and resource-efficient economy, or in other words, a higher degree of circularity.^7,9,23^ In general, the characteristics shown in this work for the TPO, both at the pilot and semi-industrial scale, are fully consistent with those found in the literature for different plant scales.^1,3,20,35,38,65^ These results therefore demonstrate the robustness of the semi-industrial plant to be considered within the seventh technology readiness level (TRL-7). Other properties shown in Table 2 also confirm the representativeness of the TPO, such as pH, TAN, and water content, which are practically the same regardless of plant size. Although TPO is transportable and storable, these characteristics are prone to possible risks of corrosion, deposits, and handling, among others.^65^

**Figure 6:**
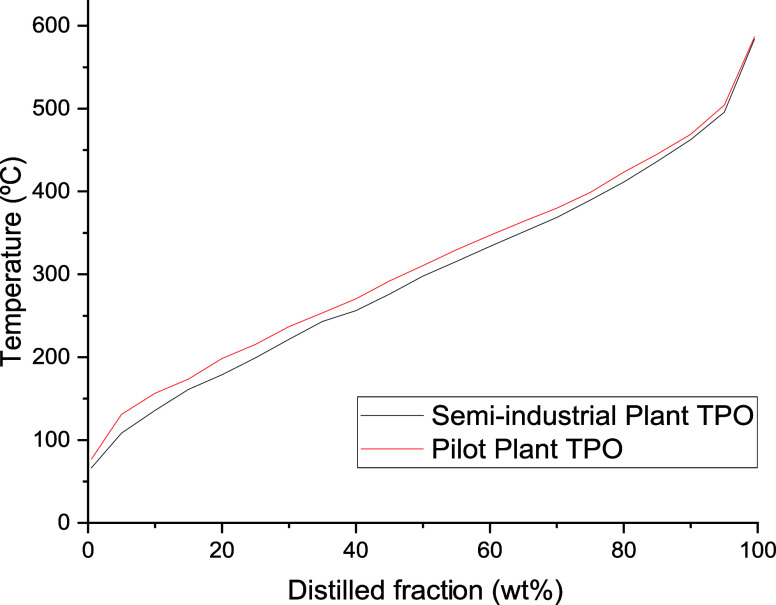
Boiling point distribution of TPO.

The boiling point distribution for TPO obtained in the two plants is also shown in Figure 6. It can be seen that both samples have the same profile, although the TPO from the pilot plant seems to contain slightly heavier compounds than that derived from the semi-industrial plant. The initial boiling point (IPB), the temperature at which 50% of the TPO is distilled (T50), and the final boiling point (FBP) are 60 and 70 °C, 290 and 300 °C, and 592 and 590 °C for the TPO produced in the pilot plant and semi-industrial plant, respectively. These high final temperatures are related to the presence of high molecular weight compounds such as polycyclic aromatic hydrocarbons (PAHs) and heterocyclic compounds, as shown elsewhere.^7,26^Figure 6 also shows that both TPOs contain a significant amount of gasoline-like, kerosene-like, and diesel-like compounds, as the cuts of these streams are within their boiling point range.^66^ It is worth noting that these data, especially those from the semi-industrial plant, are useful as a database for further simulation and design of refinery units under industrially relevant conditions with the aim of integrating the TPO into the petrochemical industry.

The concentrations of BTEX + styrene and limonene of the two TPOs are listed in Table 3. The presence of these compounds is directly related to the amount of styrene–butadiene rubber (SBR) and natural rubber (NR) in the ELTs, respectively.^6,26,67^ It is interesting to note that NR is higher in tires from heavy vehicles (27–30 wt %) than from light vehicles (15–22 wt %).^68,69^ The opposite applies to the composition of SBR. The concentration of these compounds is therefore highly dependent on the source of the ELTs. The composition of TPO is also directly related to the reaction temperature, as discussed above. In general, limonene production is maximized at low temperatures (425–450 °C), whereas single-ring aromatics are maximized at higher temperatures as they are favored by the occurrence of secondary cracking and aromatization reactions. As shown in Table 3, some minor differences can be observed between the two TPOs with regard to the concentration of the above-mentioned compounds. In this sense, BTEX and limonene account for 12.14 and 3.66 wt % and 9.37 and 4.55 wt % of the TPO produced in the pilot and semi-industrial plants, respectively. These results show once again the advantages of the temperature profile, in particular the low value in the first section of the reactor, which prevents the severe cracking of the vapors before they are condensed and collected.

**Table 3:** Composition of TPO, LF, and HF Fractions

	pilot plant			semi-industrial plant		
compound (wt%)	TPO	light fraction (LF)	heavy fraction (HF)	TPO	light fraction (LF)	heavy fraction (HF)
benzene	2.53 ± 0.03	6.15 ± 0.06	0.00 ± 0	1.54 ± 0.03	4.85 ± 0.06	0.00 ± 0
toluene	4.64 ± 0.04	9.85 ± 0.1	0.02 ± 0	3.69 ± 0.04	9.96 ± 0.1	0.03 ± 0
ethylbenzene	0.47 ± 0.01	1.08 ± 0.03	0.03 ± 0	0.56 ± 0.01	1.39 ± 0.01	0.04 ± 0
(*p*+*m*)-xylene	3.85 ± 0.1	7.08 ± 0.14	0.16 ± 0	3.13 ± 0.03	7.77 ± 0.1	0.28 ± 0
*o*-xylene	0.65 ± 0.04	2.04 ± 0.14	0.02 ± 0	0.45 ± 0.02	1.34 ± 0.1	0.04 ± 0
total BTEX	12.14 ± 0.2	26.20 ± 0.5	0.23 ± 0	9.37 ± 0.15	25.31 ± 0.4	0.39 ± 0
styrene	1.27 ± 0.05	1.00 ± 0.03	0.04 ± 0	0.55 ± 0.05	1.12 ± 0.1	0.15 ± 0
limonene	3.66 ± 0.07	10.72 ± 0.2	1.72 ± 0.03	4.55 ± 0.09	8.08 ± 0.16	1.83 ± 0.04

Table 3 also shows the concentration of BTEX + styrene and limonene for the light and heavy fractions after fractionation at 235 °C of the TPO samples. Fractionation of organic liquid mixtures by distillation is considered to be a simple strategy to carry out the initial rough separation of crude oils in such a way that compounds with similar volatility are grouped together. It can be seen that the BTEX concentration of the light fraction was more than doubled for both TPOs after fractional distillation, while in the heavy fraction it was less than 0.4 wt %. The limonene concentration in the light fraction was also very high for the two TPOs (10.72 and 8.08 wt %, respectively). The light fraction yield at 235 °C was 27% and 31% for the TPOs produced at the pilot and semi-industrial scale plants, respectively. The recovery of BTEX and limonene plays a key role in the development of ELTs pyrolysis and its integration into the petrochemical industry as these compounds are widely used in the production of various industrial and valuable products. Together with olefins (ethylene and propylene), BTEX are part of the high-value chemicals (HVCs), important building blocks for the production of plastics, resins, adhesives, cosmetics, inks, paints, pharmaceuticals, rubbers, and thinners, among others.^7,57^ Limonene also has important and diverse industrial applications. These include the production of resins and various oxygenated derivatives.

The continuous fractionation process by atmospheric distillation of TPO has recently been demonstrated by our research group under industrially relevant conditions with very promising results.^7^ In that work, the BTEX concentration in the overhead product was greater than 55 wt %, paving the way for defossilization in the chemical and petrochemical sector. Details of how the pilot distillation column for TPO was designed and operated are also given elsewhere.^8^ The distillation unit described in both works was tested and validated with TPO produced from the pilot and semi-industrial scale plants. These papers demonstrate the technical feasibility of fractionating TPO using an industrially relevant packed distillation column and provide valuable insights into the integration of pyrolysis and distillation technologies. The results reported in those papers are expected to make a significant contribution to the circular economy by effectively combining these technologies to process complex waste-based hydrocarbons, such as TPO.

### 3.4 Composition of Tire Pyrolysis Gas

The volumetric composition of the TPG (on an N_2_-free basis) is summarized in Table 4. As can be seen, both plants have very similar compositions and are particularly rich in hydrocarbons (C_*x*_H_*y*_) accounting for approximately 64 and 72 vol % for the pilot and semi-industrial plants, respectively. The most abundant C_*x*_H_*y*_ is methane (C_1_) (32–34 vol %), followed by hydrogen (24–29 vol %), which makes up more than half of the TPG. Ethane and ethylene (C_2_), propane and propylene (C_3_), *n*-butane and butadiene compounds (C_4_), and higher molecular weight C_*x*_H_*y*_ (>C_4_) are also found, with fairly similar concentrations between the two plants. Carbon monoxide as well as sulfur compounds (COS + H_2_S + CS_2_ + CH_4_S) are also observed. The latter are the main contributors to the unpleasant odor, as well as potential vectors of corrosivity and toxicity.

**Table 4:** TPG Composition in a Free N_2_ Basis

gas (vol %)	pilot plant	semi-industrial plant
H_2_	29.9 ± 0.6	24.1 ± 0.5
CH_4_	32.6 ± 2.3	34.5 ± 2.5
COx	4.6 ± 0.1	3.1 ± 0.1
C_2_	10.5 ± 0.9	13.3 ± 0.9
C_3_	3.9 ± 0.2	6.6 ± 0.4
C_4_	10.6 ± 0.9	9.0 ± 0.7
>C_4_	6.7 ± 1.3	8.9 ± 1.8
COS	0.03 ± 0	0.02 ± 0
H_2_S	1.2 ± 0.4	0.5 ± 0.2
CS_2_	0.0 ± 0	0.0 ± 0
CH_4_S	0.06 ± 0.01	0.02 ± 0
LCV (MJ/Nm^3^)	54.2 ± 2.5	52.2 ± 2.5

Therefore, gas scrubbers should be installed in large plants to prevent damage to equipment and pipelines and to meet strict environmental regulations. In fact, the semi-industrial plant includes a desulfurization system consisting of a scrubber with an NaOH solution that converts the sulfur-containing gases into soluble sodium salts. The scrubber achieves about 90% H_2_S removal efficiency using a 50% H_2_O/NaOH mixture. This solution is continuously recirculated until the pH falls below a specified level. When this occurs, the liquid in the scrubber is purged and fresh solution is introduced to bring the pH back to the desired range.

Table 4 also includes the lower calorific value (LCV) of both TPGs (52–54 MJ/Nm^3^), which shows the great potential not only to meet the energy needs of pyrolysis but also to generate electricity and/or steam, as reported elsewhere.^3,70^ It should be noted once again that the TPG produced in the semi-industrial plant is used as a fuel in an industrial burner in order to self-sustain the pyrolysis process by means of the resulting flue gases; in fact, around 70% of the TPG produced is used for this purpose. This is achieved by supplying a constant amount of air that is supplied to the burner, as the blower operates under consistent conditions. A control valve regulates the TPG supply to the burner, allowing the system to automatically adjust the valve opening and the amount of TPG supplied once the temperature is set. As a result, the TPG cleaning system worked efficiently and avoided the risks associated with corrosion during operation. The semi-industrial plant also represents an excellent example of energy integration and therefore energy efficiency, as the energy required by the process, namely the energy for pyrolysis, is supplied by the TPG. Future integration with refinery and petrochemical units such as distillation can also bring significant benefits by replacing part of the operation with TPG.

### 3.5 Properties of the Raw Recovered Carbon Black

It is well-known that RRCB contains different grades of CB and is therefore considered to be a complex mixture of many and heterogeneous carbon particles. It also includes inorganic elements and exogenous carbonaceous deposits.^10,71,72^ The recovery and use of RRCB is particularly important not only because of the huge and growing CB market,^73^ but also because of the carbon footprint associated with its production (2.4 kg CO_2_/kgCB).^31^ The RRCB therefore has particular interest in the achievement of a sustainable and circular economy. Table 5 summarizes some of the characteristics of the RRCBs produced in the pilot and semi-industrial plants. It can be seen that comparable values were found, which once again confirms the robustness of the semi-industrial scale plant.

**Table 5:** RRCB Characterization

analysis	pilot plant	semi-industrial plant
Proximate, dry basis (wt %)		
ash (wt %)	14.7 ± 1.5	16.6 ± 1.7
volatile matter (wt %)	1.6 ± 0.1	4.6 ± 0.4
fixed carbon (wt %)	83.8 ± 0.4	76.4 ± 0.4
Ultimate, dry basis (wt %)		
carbon	84.5 ± 0.4	80.1 ± 0.4
hydrogen	0.4 ± 0.0	1.7 ± 0.1
nitrogen	0.3 ± 0.0	0.4 ± 0.0
sulfur	2.8 ± 0.3	3.3 ± 0.3
Calorific value		
HCV (MJ/kg)	29.6 ± 1.0	27.8 ± 1.0
Others		
S_BET_ (m^2^/g)	54.0 ± 0.3	56.9 ± 0.3
transmittance of toluene extract (%)	40.5 ± 1.2	0.12 ± 0.01

However, there is a noticeable difference between the volatile matter levels (1.6 wt % for the pilot plant and 4.6 wt % for the semi-industrial plant). Volatile matter is generally associated with the presence of carbonaceous deposits in the RRCB coming from nondevolatilized rubber and/or condensed hydrocarbon compounds in the surface. The transmittance of the toluene extract obtained by the ASTM D1618 method is a representative indicator of the presence of these carbonaceous deposits on the RRCB, or, in other words, of the organic impurity content. In this case, the transmittance of the toluene extract was lower for the solid obtained in the semi-industrial plant (0.1%) than that for the solid obtained in the pilot plant (40%), as expected due to the higher volatile content of the sample. It is desirable to keep the volatile matter as low as possible to improve the quality of the RRCB. However, in industrial-scale plants, adequate control of the reactor to prevent the occurrence of this volatile matter in the RRCB is more complex than that in laboratory and pilot scale plants. This challenge could be overcome by intensive milling, as the volatile matter tends to both break and separate from the CB matrix when ground into powder, resulting in dust-free, free-flowing rCB granules. This reduces the size of the fused agglomerates, and the interactions between the CB particles are reduced, ultimately improving the quality of the material, such as dispersibility when used in rubber formulations.

Based on the above results, the ASTM D8178 standard distinguishes between RRCB and rCB, as mentioned in Section 1, in order to differentiate between those with low and high reinforcing properties, respectively. In addition, the mechanical grinding of RRCB is expected to form chemical bonds that provide new oxygenated functional groups whose nature and relative quantity can change depending on the reaction time, as reported elsewhere.^74^ The presence of these functional groups after milling is expected to increase the surface activity of the rCB; resulting in stronger bonds are formed when used, for example, in polymer formulations.^71^ The milling step, as well as palletization to improve handling and shipping, have been used by industrial companies involved in the pyrolysis of ELTs in order to provide rCBs with a comparable performance to N300, N500, N600, and N700 CB grades.^75^ The BET surface area of the RRCB produced in both plants was similar to values of 54 and 57 m^2^/g. These values are in the range of the commercial CB. The versatility of the auger reactor configuration is highlighted with regard to the use of different potential temperature profiles, in particular the high temperature in the tail section of the reactor as a strategy to minimize the presence of exogenous carbon deposits in the RRCB. Although this plan is currently being confirmed by the accumulation of hours in the semi-industrial plant, the results obtained so far confirm the potential of this strategy to maximize the benefits of all the products obtained.

### 3.6 Scale-Up Analysis and Product Consistency in Tire Pyrolysis

This section deals with the scaling up of the pyrolysis process from pilot to semi-industrial scale, with a focus on the consistency of yields and characteristics across different particle sizes.

- Confirmation of scale-up: the observed consistency in product composition indicates successful scale-up of the pyrolysis process from laboratory to semi-industrial scale. This suggests that the operating parameters, such as temperature profiles and residence times, are sufficiently robust to accommodate variations in particle size without significantly altering the final product composition.
- Mass and energy transfer: in an auger reactor, efficient mass and energy transfer can be achieved due to the inherent mechanical forces that enhance particle mixing. This helps to maintain uniform temperature distribution and consistent reaction conditions throughout the reactor. The continuous movement of the particles, regardless of their size, exposed them to a constant thermal environment, resulting in successful pyrolysis reactions.
- The thermal conductivity of the feedstock in the reactor plays a crucial role in ensuring uniform heat distribution. Although larger particles may take longer to heat up, the overall heat transfer mechanisms in the auger reactor are satisfactorily controlled by the feed mass rate to ensure that both the interior and surface of the particles reach the temperatures required for pyrolysis.
- Residence time: optimizing residence time for both vapors and solids is critical for effective pyrolysis. In this work, the residence time of the vapors was optimized to avoid the occurrence of secondary reactions, which was ultimately controlled by the feed mass rate. Auger reactors have the advantage that the residence time of the solids can be easily controlled by varying the rotation speed, ensuring complete pyrolysis of both small and large particles. The auger reactor design facilitates uniform residence time distribution, which contributes to the observed similarity in product yields.

Thus, the scale-up process maintained the integrity of the pyrolysis reaction across different particle sizes, confirming the robustness and efficiency of the process. In addition, it is important to consider other types of technologies available at an industrial level in order to provide a more realistic view of the processes being carried out. In the context of pyrolysis of ELTs, fixed bed reactors, particularly moving bed technology and rotary kiln reactors, are the most commonly used and accessible options. When these results are compared with other industrial plants, several observations can be made. Pyrum Innovations AG using a moving bed technology at TRL-9 (5000 ton/y for reactor) produces 31, 44, and 25 wt % of TPO, RRCB and TPG, respectively^76^ This indicates a slightly lower TPO yield but a higher RRCB yield compared to the auger technology described in this work. These differences can be directly related to the reactor design and operating conditions as the volatile residence time is much higher with moving bed technology.

On the other hand, studies relating to rotary kiln technology^17^ report yields of 15 ± 3 wt % TPG, 40 ± 4 wt % TPO, and 45 ± 4 wt % RRCB at approximately 450 °C using mixtures of different types of ELTs with a particle sizes in the range of 5–20 mm. These results are in close agreement with our findings, particularly in TPO and TPG, although the RRCB yield is slightly higher in the rotary kiln. Another relevant study^1^ describes a semi-industrial prototype (TRL-7) operating at 550 °C using 50 to 300 mm particles, achieving yields of 14.5 ± 2 wt % TPG, 37.5 ± 1 wt % TPO, and 48.0 ± 2 wt % RRCB. That prototype, like the auger reactor, operates at a similar TRL level and has comparable yields in the TPO and TPG but again has a higher RRCB.

The consistent yields across these different reactor types: moving bed, rotary kiln, and auger, suggest robustness in the operating parameters such as temperature profiles and residence times. The variation in RRCB and TPO yields can be attributed to differences in reactor design and the efficiency of mass and energy transfer mechanisms. In particular, the continuous mixing and movement of the auger reactor facilitate uniform thermal conditions, which may contribute to its distinctive yield distribution. These findings underline the effectiveness of our TRL-7 auger reactor in the wider context of industrial pyrolysis technologies.

### 3.7 Technical Issues and Concerns

Details of different reactor designs for the pyrolysis of ELTs, including operating principles, and even throughputs are found elsewhere.^77^ Accordingly, fixed beds and rotary kilns are currently the most widely used technologies worldwide, and both are found on an industrial scale with several suppliers.^5^ However, the auger pyrolyzer detailed in this work clarifies the exogenous risks associated with scale and provides key information to consider this technology as reliable, being possible included among the different options for pyrolysis of ELTs on an industrial scale. Although the semi-industrial plant has been demonstrated to be capable of working flawlessly and according to the expected pattern based on the pilot plant (TRL-5), some concerns and issues are raised in order to move forward a higher level of technological maturity.

- During process control, special attention should be focused on pressure, especially at the vapor outlet. No variations in this parameter is an essential indicator that the pyrolysis process is being carried out correctly. The ducts that evacuate the gaseous fraction can be a key factor in ensuring long-term operation, and special attention must be paid to during maintenance, cleaning and overhaul work.
- It is highly recommended that the ELTs particles are free of steel and textile contamination in order to avoid clogging and ensure the correct operation.
- The presence of corrosive agents such as sulfur-containing compounds in both TPO and TPG makes mandatory the use of stainless steel in pipelines and other mechanical elements of the plant which could be in direct contact with these compounds.
- The presence of different thermocouples and pressure sensors along the reactor certainly provides more information about what is happening during the process, making control procedures and decisions more efficient and timely.

## 4 Conclusions

This work shows a detailed comparison of the yields and characteristics of the resulting products derived from pyrolysis of end-of-life tires between a pilot prototype (4 kg/h) and a semi-industrial plant (400 kg/h) both using the auger technology. The pilot prototype, ranked within the fifth technology readiness level (TRL-5), has been operated and revamped over time for the Environmental Research Group of the Instituto de Carboqumica (ICB-CSIC) and has served to provide key data for setting technical specifications of the semi-industrial plant. In this sense, the yields and properties of tire pyrolysis oil, tire pyrolysis gas, and raw recovered carbon black are notoriously similar between the two plants. These resemblances support the reliability and robustness of the semi-industrial plant to be considered within the seventh level of technology readiness (TRL-7).

## Symbols

symbol: meaning
BTEX: benzene, toluene, ethyl-benzene, xylenes
CB: carbon black
CO_2_: carbon dioxide
CH_4_: methane
CH_4_S: methyl mercaptan
CO_*x*_: carbon oxides (e.g., CO, CO_2_)
C2, C3, C4: hydrocarbons with two, three, and four carbon atoms
>C4: higher C_4_ hydrocarbons
COS: carbonyl sulfide
CS_2_: carbon disulfide
CSIC: Spanish Council for Scientific Research
ELTs: end-of-life tires
FBP: final boiling point
FCC: fluid catalytic cracking
FID: flame ionization detector
GC: gas chromatograph
H_2_: hydrogen
H_2_S: hydrogen sulfide
H-1, 2, 3: hopper 1, 2, 3
HF: heavy Fraction
HHV: higher heating value
IPB: initial boiling point
LF: light fraction
LHV: lower heating value
LPG: liquefied petroleum gas
ICB: Instituto de Carboquímica
M-1: motor 1
NaOH: sodium hydroxide
N_2_: nitrogen
NR: natural rubber
PAHs: polycyclic aromatic hydrocarbons
pH: measure of acidity or alkalinity
PI: pressure indicator
PTR: Parque Tecnológico de Reciclado
rCB: recovered carbon black
RRCB: raw recovered carbon black
SBR: styrene–butadiene rubber
T: temperature (typically in degrees Celsius)
TAN: total acid number
TCD: thermal conductivity detector
TIC: temperature indicator controller
TPG: tire pyrolysis gas
TPO: tire pyrolysis oil
TRLs: technology readiness levels
TTs: truck tires
